# The role of voltage-gated potassium channels in the regulation of mouse uterine contractility

**DOI:** 10.1186/1477-7827-5-41

**Published:** 2007-11-02

**Authors:** Ryan C Smith, Marisa C McClure, Margaret A Smith, Peter W Abel, Michael E Bradley

**Affiliations:** 1 Department of Pharmacology, Creighton University Medical Center, Omaha, USA, NE 68178

## Abstract

**Background:**

Uterine smooth muscle cells exhibit ionic currents that appear to be important in the control of uterine contractility, but how these currents might produce the changes in contractile activity seen in pregnant myometrium has not been established. There are conflicting reports concerning the role of voltage-gated potassium (Kv) channels and large-conductance, calcium-activated potassium (BK) channels in the regulation of uterine contractility. In this study we provide molecular and functional evidence for a role for Kv channels in the regulation of spontaneous contractile activity in mouse myometrium, and also demonstrate a change in Kv channel regulation of contractility in pregnant mouse myometrium.

**Methods:**

Functional assays which evaluated the effects of channel blockers and various contractile agonists were accomplished by quantifying contractility of isolated uterine smooth muscle obtained from nonpregnant mice as well as mice at various stages of pregnancy. Expression of Kv channel proteins in isolated uterine smooth muscle was evaluated by Western blots.

**Results:**

The Kv channel blocker 4-aminopyridine (4-AP) caused contractions in nonpregnant mouse myometrium (EC50 = 54 micromolar, maximal effect at 300 micromolar) but this effect disappeared in pregnant mice; similarly, the Kv4.2/Kv4.3 blocker phrixotoxin-2 caused contractions in nonpregnant, but not pregnant, myometrium. Contractile responses to 4-AP were not dependent upon nerves, as neither tetrodotoxin nor storage of tissues at room temperature significantly altered these responses, nor were responses dependent upon the presence of the endometrium. Spontaneous contractions and contractions in response to 4-AP did not appear to be mediated by BK, as the BK channel-selective blockers iberiotoxin, verruculogen, or tetraethylammonium failed to affect either spontaneous contractions or 4-AP-elicited responses. A number of different Kv channel alpha subunit proteins were found in isolated myometrium from both nonpregnant and term-pregnant mice, and one of these proteins – Kv4.3 – was found to disappear in term-pregnant tissues.

**Conclusion:**

These findings suggest a role for Kv channels in the regulation of uterine contractility, and that changes in the expression and/or function of specific Kv channels may account for the functional changes seen in pregnant myometrium.

## Background

The smooth muscle of the uterus changes remarkably during pregnancy. Despite the presence of spontaneous, myogenic contractility, the myometrium must remain quiescent as it is stretched to an extraordinary degree during pregnancy, but suddenly develop the highly organized and powerful contractions characteristic of labour. Myometrial cells in pregnant animals express increased densities of oxytocin receptors and gap junctions which are known to play important roles in uterine contraction[1]; these changes appear to be essential but not sufficient for the onset of labouring contractions. The signals which trigger labour are not known. Our overall goal is to determine the changes in fundamental properties which must occur in uterine smooth muscle cells during the transition into labour.

The biochemical mechanisms associated with contraction in the myometrium are reasonably well understood. There is also a great deal of evidence that changes in ionic conductances across the plasma membrane are involved in the regulation of spontaneous and agonist-evoked contractions in uterine myocytes from both pregnant and nonpregnant animals [2-4]. Spontaneous contractions in uterine myocytes are associated with and probably arise from gradual depolarisations of resting membrane potential [3], and there is evidence that maximum resting membrane potentials decrease in uterine smooth muscle cells in women and rats during the course of pregnancy [5-7]. However, our understanding of the roles played by specific ion currents in the regulation of contractile activity is poor, and the molecular identity of the channels which underlie these currents is in most cases not known. Changes in electrical currents in uterine myocytes may be important points of regulation of contractility in the transition between the quiescent and labouring uterus at the end of gestation [2,3].

Action potentials in uterine myocytes result from activation of voltage-operated calcium channels; membrane depolarisations are halted and reversed by the inactivation of these channels as well as the concurrent activation of outward currents carried by potassium ions [8,9]. Several types of potassium channels are known to be present in myometrium, including the large-conductance, calcium-activated potassium (BK) channel [10,11], the ATP-sensitive potassium (K_ATP_) channel [12,13], and at least one inward rectifier [14]. There is also some evidence for potassium currents in myometrium which are voltage gated [15], including evidence for the presence of Kv channels which are assumed to consist of multimers of α subunits which are known to form the pore region of these voltage-gated channels [16-19,16]. [19,20]. Large-conductance, calcium-activated potassium channels have been shown to be involved in the repolarisation of cell membranes during action potentials. In uterine smooth muscle cells the currents responsible for setting resting membrane potential – a key component of regulation of activity in other excitable cells – are not known. It has been suggested that BK, at least in human myometrium, is the current responsible for regulating electrical (and thus contractile) activity [10], but experiments performed upon rat myometrium have produced contradictory findings which argue against a role for BK in the regulation of contractility in either pregnant or nonpregnant myometrium[21].

We now describe a large contractile response to the Kv channel-selective blocker 4-aminopyridine (4-AP) in myometrium; this response is not mediated by BK channels, the endometrium, or nerves. We also demonstrate the expression in both nonpregnant and pregnant mouse myometrium of a number of Kv channel α subunit proteins, including several types known to be sensitive to 4-AP. Remarkably, contractile responses to 4-AP disappear in pregnant mouse myometrium, and this finding is correlated with the loss in expression of the Kv4.3 α subunit protein. The presence of a contractile response to 4-AP as well as a number of Kv channel α subunit proteins in mouse myometrium suggests a role for Kv channels in the regulation of myometrial function. Furthermore, the disappearance of Kv4.3 expression and of responses to 4-AP and phrixotoxin-2 in pregnant myometrium may indicate a specific loss of Kv channel function at the point when the myometrium is being prepared for labour.

## Methods

### Preparation of uterine tissues and contraction experiments

Contractile studies were performed on uterine tissues obtained from virgin 8–25 week-old or timed (7–20 days following a single 12 hour caging with a proven male) pregnant mice, with term-pregnancy being defined as day 20 of gestation (P20); female mice were of the C57BL/6J strain (Jackson Laboratories, Bar Harbor, ME), but males were CF-1 mice obtained from Charles River Laboratories, Wilmington, MA. Animals were sacrificed by cervical dislocation according to protocols approved by the Creighton Institutional Review Board; uterine horns were removed and immediately placed into ice-cold contraction buffer consisting of the following (in mM): NaCl, 120; KCl, 5.0; KH_2_PO_4_, 0.59; Na_2_HPO_4_, 0.60; MgCl_2_, 2.6; dextrose, 20.0; tris (hydroxymethyl) amino-methane, 25.0; CaCl_2_, 1.8. Uterine horns were opened by cutting along the mesometrial border while immersed in ice-cold oxygenated buffer, pinned serosal-side down on a bed of Sylgard^® ^(Dow Corning, Midland, MI) and strips approximately 10 mm × 5 mm × 2 mm of full-thickness (endometrium plus myometrium) uterine tissue were sliced from the centre of the horn with the long axis of the strip parallel to the long axis of the horn *in situ*. In experiments utilizing isolated (endometrium-free) myometrium, a small incision was made through the endometrium at one end of each horn and the endometrium was carefully peeled away from the underlying smooth muscle layers by the use of fine-tipped forceps; both outer longitudinal and inner circular muscle layers remained in these preparations, and each of these layers was found to consist of cells approximately 20 layers thick. Tissue strips were attached to TRN 20 g isometric force transducers (Kent Scientific, Litchfield, CT) by silk thread in 20 ml water-jacketed organ baths to favour recording from the longitudinal muscle layer; transducer voltages were amplified and converted to digital signals by an ACJr^® ^A/D board mounted within a computer system running the Workbench^® ^data acquisition system (Strawberry Tree, Inc., Sunnyvale, CA). Strips were maintained at 37°C with constant aeration with 100% O_2_. Tissues were loaded with an initial tension of 0.25 grams and challenged with maximal concentrations of acetylcholine (100 μM) or oxytocin (1 μM) during the course of a 30–60 minute equilibration period. In experiments in which the Kv4.2/Kv4.3 blocker phrixotoxin-2 was used, a specially-designed and -built micro organ bath assembly was used so that tissues could be mounted in very small (200 μL), vertically-oriented chambers milled out of cast acrylic in order to reduce the amount of phrixitoxin-2 required; in this apparatus tissues were constantly superfused with warm, oxygenated contraction buffer.

Noncumulative concentration-response relationships were developed in a manner which allowed each tissue strip to serve as its own control. Spontaneous contractile activity for 5 minutes immediately preceding each drug challenge was quantified and subtracted from values obtained from subsequent 5 minute drug challenges in order to control for differences in spontaneous contractile activity among different tissue strips, and to control for changes in spontaneous contractility that might occur during the course of each experiment. Pretreatment of tissues with maximal concentrations of toxins was performed for a minimum of 10 minutes. Tissues were allowed to relax for at least 10 minutes after each agonist stimulation, during which tensions returned to baseline values. Addition of 4-AP at the highest concentrations used did not alter the pH of the solution in the organ baths. Dimethylsulfoxide was the solvent for verruculogen and did not reach concentrations in organ baths higher than 0.01% by volume; all other solutions were created using contraction buffer as the solvent.

### Data analysis

Contractile responses were quantified by integration of the area underneath the contractile record by software specifically written for this purpose. Spontaneous contractile activity was quantified and subtracted from all drug-elicited responses. Unless stated otherwise, values for force of contraction are expressed as mg tension generated, ± one standard error of the mean (S.E.M.). Population sample numbers (*n*) indicate the number of animals used, and not simply number of tissues. Comparison of mean responses to maximal concentrations of 4-AP were compared for all time points by means of a one-way ANOVA followed by Tukey's post-hoc test.

### Western blotting

Samples of isolated, endometrium-free uterine smooth muscle (approx. 500 mg/sample) were placed into a 1× sample buffer consisting of 125 mM Tris-HCl, 2% SDS, 5% glycerol, 0.003% bromophenol blue, and 1% 2-mercaptoethanol; samples were homogenized using a Duall^® ^glass-glass tissue grinder until completely disrupted. After heating samples for 5 minutes at 95°C, precipitates were removed by centrifugation. Separation of proteins was performed by means of loading polyacrylamide gels containing 8% acrylamide and stacking gels made to contain 4.75% acrylamide with 20 μg of protein in each lane; these gels were built using a BioRad^® ^mini-gel apparatus in order to accomplish protein separations within 1 hour at a constant voltage of 200 V. High-range, pre-stained molecular weight markers were used to provide an indication of apparent molecular weights following transfer onto polyvinylidene fluoride (PVDF) membranes. Following separations, gels were immediately used as donors for transfer of proteins to PVDF membranes by means of a semi-dry transfer apparatus; at room temperature this procedure took 90 minutes at a constant current of 0.8 mA/cm^2^. Membranes were blocked with 5% (w/v) nonfat milk plus 0.05% Tween 20 and sodium azide at 0.02% (w/v) in Tris-saline solution containing 150 mM NaCl and 10 mM tris (hydroxymethyl) amino-methane at 4°C overnight with rocking. Incubations with primary antibodies (all purchased from Alomone Labs, Jerusalem: Kv1.1 (cat. #AN-03), Kv1.2 (cat. #AN-04), Kv1.3 (cat. #AN-03), Kv1.4 (cat. #AN-06), Kv1.5 (cat. #AN-04), Kv1.6 (cat. #AN-03), Kv4.2 (cat. #AN-04) and Kv4.3 (cat. #AN-04)) at dilutions of 1:200-500 were performed in Tris-saline solution containing nonfat milk at 0.05% (w/v), Tween 20 at 0.05% (v/v) and sodium azide at 0.02% (w/v) for 1.5 hours at room temperature with rocking. After extensive washing, membranes were incubated with secondary antibody (goat anti-rabbit, 1:15,000, Southern Biotech) in a Tris-saline solution containing nonfat milk at 0.05% (w/v) and Tween 20 at 0.05% for 1.5 hours at room temperature. Secondary antibodies were conjugated to horseradish peroxidase; following washing of membranes, light-generating reactions were initiated by incubation of filters in a solution containing ECL^® ^reagent (Pharmacia) and filters were exposed to X-ray films. Band densities were quantified by means of the Molecular Analyst^® ^program, normalized to the darkest band present on each film, and compared by one-way ANOVA.

## Results

### 4-AP contracts nonpregnant myometrium

**Figure 1:**
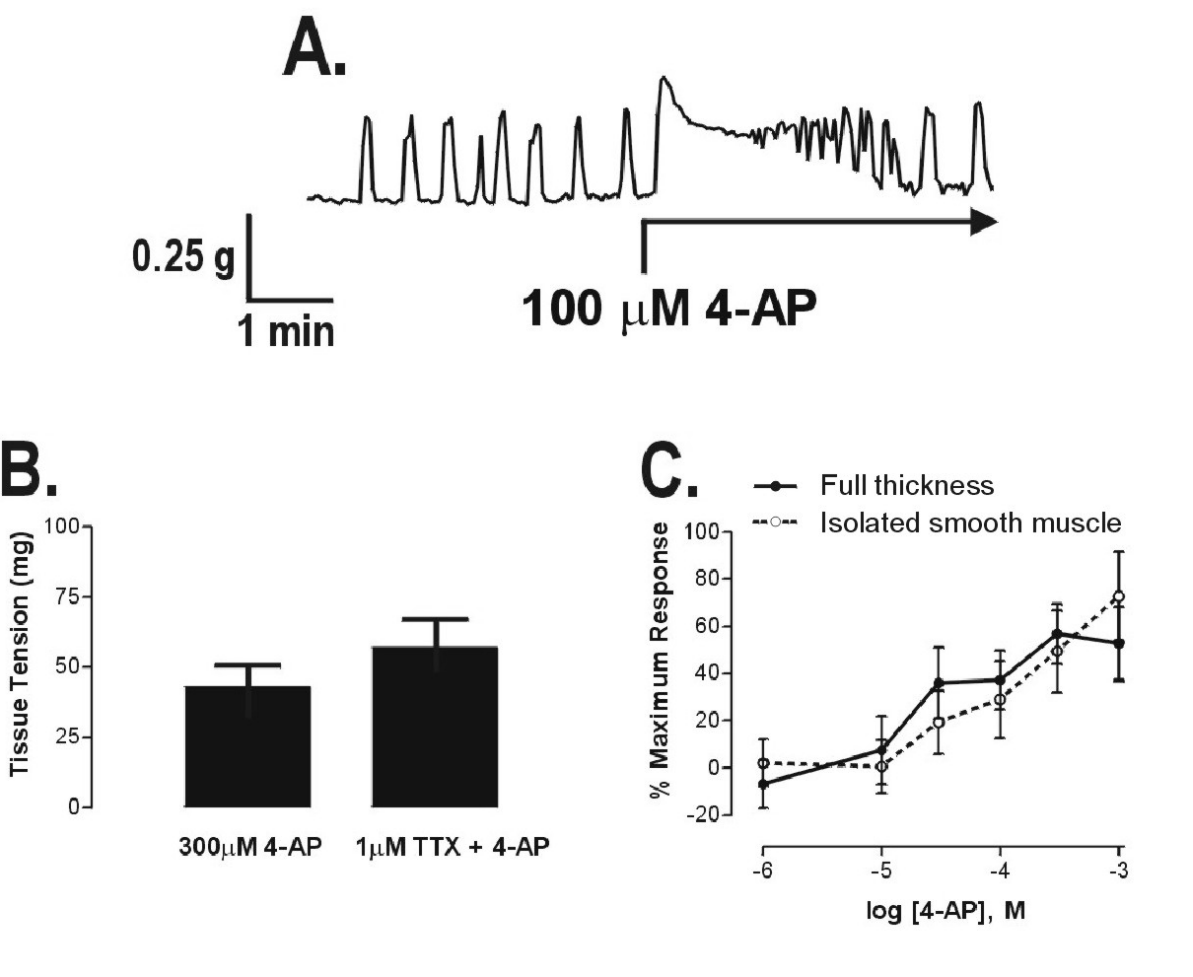
**4-AP contracts the nonpregnant (NP) mouse myometrium**. (A) Representative contractile recording of the response to 4-AP in nonpregnant mouse myometrium. Horizontal component of arrows denotes duration of 4-AP incubation. (B) Pretreatment (15 min) of nonpregnant myometrial tissue strips with 1 μM tetrodotoxin (TTX) failed to alter the contractile response to 4-AP (*n *= 4). (C) Concentration-response relationships of full-thickness tissue strips (filled circles, *n *= 5) and isolated, endometrium-free myometrium (open circles, *n *= 4); data are responses in nonpregnant mice expressed as a mean percentage (± S.E.M.) of the maximal response elicited by 4-AP in each tissue strip, minus the spontaneous activity immediately preceding each addition of 4-AP.

Mouse myometrium exhibited both spontaneous and agonist-elicited contractions *in vitro*. Contractile responses to 4-AP in nonpregnant samples were characterized by an increase in baseline tension and an increase in the frequency of phasic activity (Figure 1.a), and responses to maximal concentrations of 4-AP were smaller than those seen in response to maximal concentrations of either acetylcholine or oxytocin. Upon washout of contractile agents, tissue activities resembled those present immediately prior to agonist addition, and no cumulative effects of drug treatment were observed during the course of experiments. Nerves were not involved in contractile responses to 4-AP, as incubation of tissues with the voltage-activated sodium channel blocker tetrodotoxin (1 μM, 15 minutes) had no significant effect (Figure 1.b), and responses to 4-AP were not significantly altered by storage of either full-thickness tissues or isolated myometrium at room temperature for 18 hours (data not shown), during which it is assumed that severed nerves remaining in the preparations would have lost their ability to function. The endometrium was not required for 4-AP-elicited contractions in nonpregnant myometrium, as a comparison of concentration-response curves between full-thickness tissue strips and isolated myometrium (endometrium removed) revealed no significant differences in 4-AP potency (EC_50 _≈ 54 μM) or efficacy (Figure 1.c).

### Lack of a role for BK in either spontaneous- or 4-AP-elicited contractions

**Figure 2:**
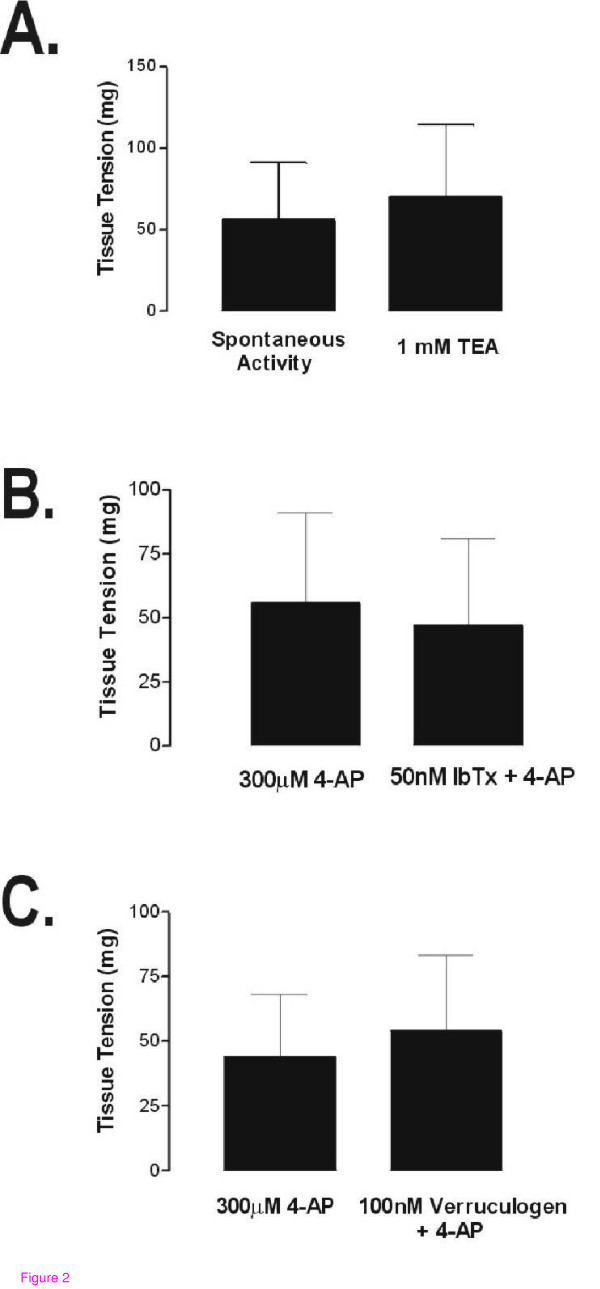
**Large-conductance, calcium-activated potassium channel is not involved in either the 4-AP response or spontaneous activity in nonpregnant mouse myometrium**. (A) Tissue strips from nonpregnant mice were challenged with 1 mM of the BK blocker TEA. There were no significant differences found between tissue strip contractility in the presence of TEA when compared to spontaneous (no TEA) activity. (B and C) Tissue strips from nonpregnant mice were challenged with 300 μM 4-AP for 5 min, washed for 10 minutes, and then incubated with either iberiotoxin (IbTX, 50 nM, panel B) or verruculogen (100 nM, panel C) for 10 minutes prior to re-challenge with 300 μM 4-AP; the amount of spontaneous contractile activity present in each sample was subtracted from the total activity seen in the presence of either 4-AP or 4-AP plus BK blocker to account for any changes in spontaneous activity. Mean responses in the presence of toxin (A and B) or TEA (C) were not significantly different from those seen in the absence of either toxin or TEA. Results are presented as means ± S.D.; *n *= 4 for each panel.

Addition of the BK blocker tetraethylammonium (TEA) at concentrations as high as 1 mM to spontaneously-active myometrium had no effect on either the frequency or amplitude of spontaneous contractions (not shown), nor did it affect the total amount of tension generated by these tissues (Figure 2.a). Contractile responses to 4-AP were also not mediated by BK channels, as treatment of tissues with maximal concentrations of either of the BK channel blockers iberiotoxin or verruculogen had no effect upon subsequent responses to 4-AP (Figure 2.b, c); neither iberiotoxin nor verruculogen had any effects on spontaneous activity alone (*i.e*., when added during the preincubation periods, data not shown). As indicated in the legend to Figure 2, spontaneous activity has been mathematically removed from the values indicating responses to 4-AP in panels B and C so as to avoid any contribution of changing spontaneous activity levels to the assessment of the effects of either 4-AP or blockers of BK.

### Loss of 4-AP-induced contractions in pregnancy

**Figure 3:**
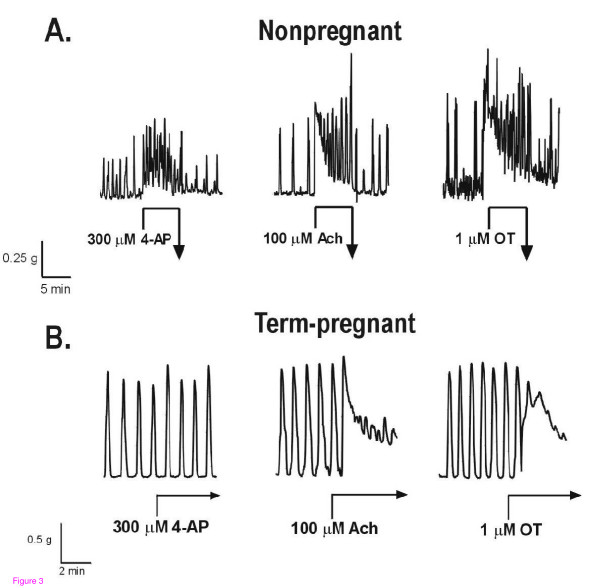
**Disappearance of 4-AP responses in pregnant mouse myometrium**. Representative contractile recordings are from nonpregnant (panel A) and term-pregnant (P20) mouse myometrium (panel B). Horizontal component of arrows denotes duration of each drug incubation. All 3 recordings in each panel were obtained from the same tissue strip and are representative of results obtained from 5 nonpregnant and 5 term-pregnant mice. Panel A: Contractile responses to maximal concentrations of 4-AP, acetylcholine (Ach), and oxytocin (OT) in nonpregnant mouse myometrium. Panel B: Contractile responses to Ach and OT are shown in contrast to the absence of a contractile response to 300 μM 4-AP in term-pregnant mouse myometrium.

**Figure 4:**
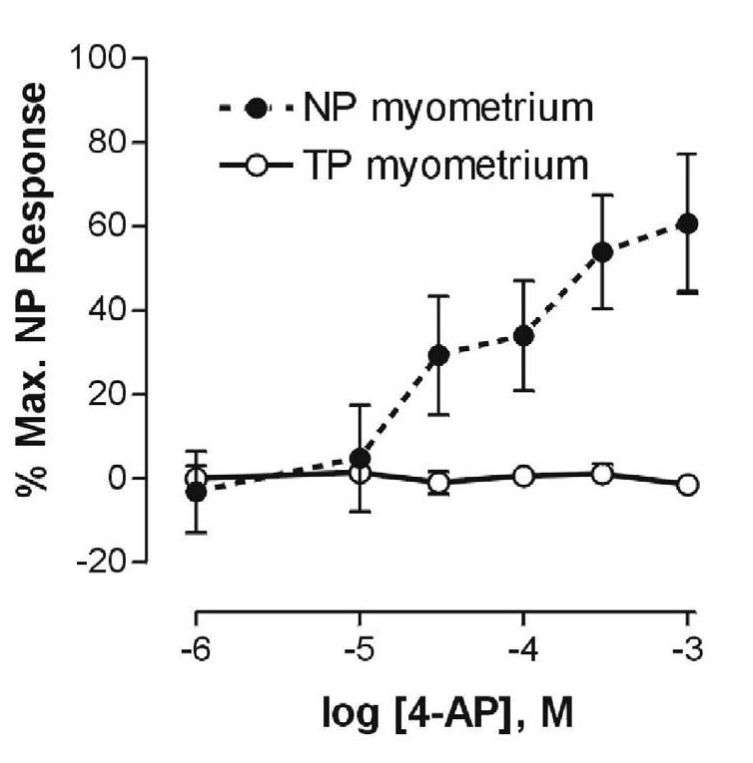
**4-Aminopyridine concentration-response relationships in nonpregnant (NP) and term-pregnant (P20) mouse myometrium**. Non-cumulative additions of 4-AP at concentrations above 10 μM produced contractions in nonpregnant tissues (filled circles, *n *= 5) but not in term-pregnant tissues (open circles, *n *= 5). Results are presented as the mean percentage of the maximal response to 4-AP in nonpregnant tissue strips, ± S.E.M.

4-Aminopyridine, while capable of eliciting contractions in nonpregnant myometrium at relatively low concentrations, did not contract myometrium obtained from term-pregnant mice (Figure 3.b, left-most recording) even when present at concentrations as high as 1 mM (Figure 4, open circles); term-pregnant tissues remained, however, fully responsive to acetylcholine or oxytocin (Figure 3.b).

### Expression of Kv channel proteins

**Figure 5:**
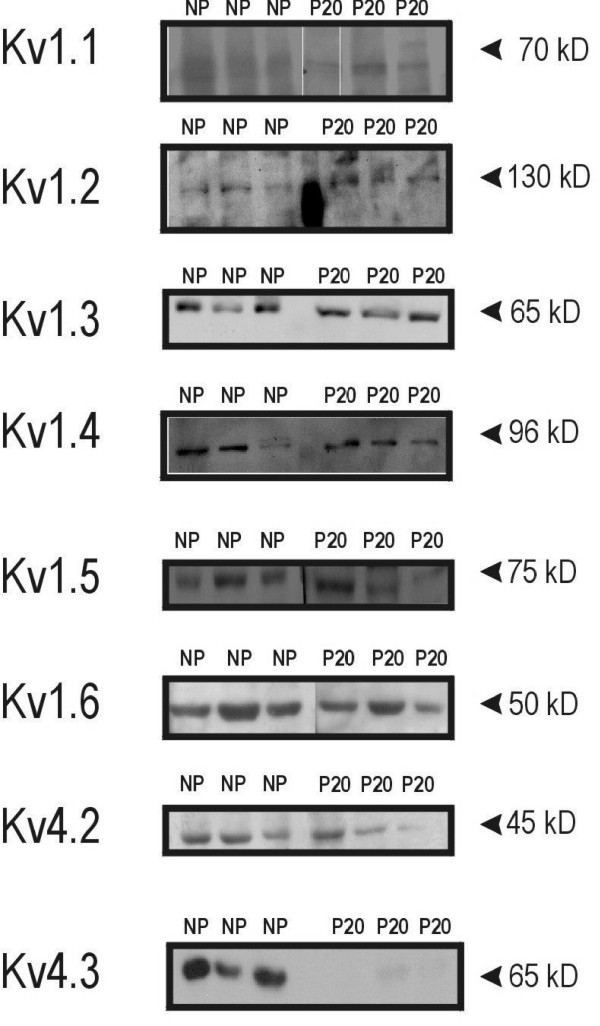
**Kv channel α subunit proteins are expressed in mouse myometrium**. Results obtained from Western blots demonstrating the presence of immunoreactive bands for Kv1.1, Kv1.2, Kv1.3, Kv1.4, Kv1.5, Kv1.6, and Kv4.2 α-subunits in myometrium from nonpregnant (NP) and term-pregnant (P20) mice. Kv4.3 α-subunits were detected in the NP samples, but were undetectable in the term-pregnant mouse myometrium. Apparent molecular weights of each protein species are indicated at right. Different lanes contain samples from different animals, and each lane was loaded with 20 μg protein; 4–7 animals were assayed for each Kv channel α subunit protein.

Because 4-AP is a well-known blocker of Kv channels, we sought to determine whether any Kv channel proteins are present in mouse myometrium. Several types of Kv channel α subunit proteins were found to be expressed in isolated, endometrium-free uterine smooth muscle from both pregnant and nonpregnant mice (Figure 5). No consistent differences in expression between nonpregnant and term-pregnant tissues were seen for Kv1.1, Kv1.2, Kv1.3, Kv1.4, Kv1.5, Kv1.6, or Kv4.2. However, while Kv4.3 was expressed at relatively high levels in nonpregnant myometrium, we were essentially unable to detect this protein by Western blot in myometrial samples from term-pregnant mice (Figure 5, bottom panel).

### Loss of Kv4.2/Kv4.3 responses in pregnancy

**Figure 6:**
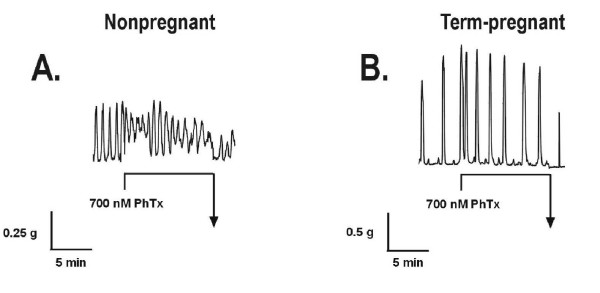
**Phrixotoxin-2 (PhTx) contracts the nonpregnant but not the term-pregnant myometrium**. Representative contractile recordings are from nonpregnant (panel A) and term-pregnant (panel B) mouse myometrial tissue strips. Horizontal component of arrows denotes duration of each drug incubation.

**Figure 7:**
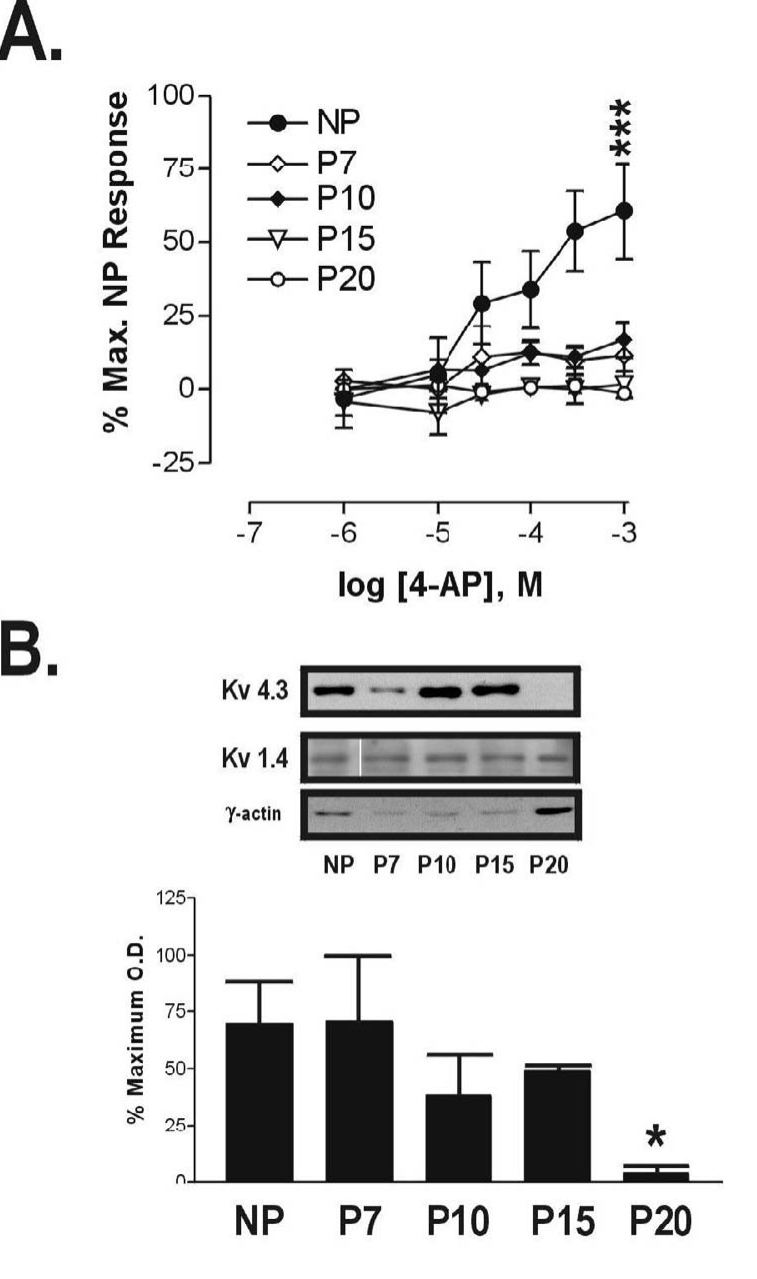
**Time course of loss of 4-AP responsiveness and Kv4.3 expression in pregnant mouse myometrium**. Myometrial samples were obtained from nonpregnant (NP, n = 5), 7 days pregnant (P7, n = 3), 10 days pregnant (P10, n = 3), 15 days pregnant (P15, n = 4) and term-pregnant (P20, n = 5) mice. Panel A: Results from non-cumulative concentration-response experiments are presented as mean percentages of the maximal response to 4-AP in NP tissues. Panel B: Densitometry of Kv4.3 expression in the same samples evaluated in panel (A) and typical Western blot results demonstrating expression of Kv4.3 as well as Kv1.4 and γ-actin to confirm equal protein loading. Band densities are expressed in graph as a percentage of the maximal band density on each film; asterisks denote a significant difference (P = 0.01) between average NP and term-pregnant sample band densities.

In an attempt to correlate the loss of 4-AP responses with the loss of Kv4.3 expression in pregnant myometrium, we used the Kv4.2/Kv4.3-specific blocker phrixotoxin-2 in contraction experiments on both nonpregnant and term-pregnant tissues. After determining the concentration at which phrixotoxin-2 elicits a maximal contraction in mouse myometrium (700 nM, data not shown), we found that while phrixotoxin-2 consistently elicited contractile responses in nonpregnant tissues which were characterized by an increase in baseline tension without any significant increase in frequency of contractions (Figure 6.a), it failed to produce contractions in tissues from term-pregnant mice (Figure 6.b) even when used at concentrations as high as 1.3 μM; phrixotoxin-2 effects were not observed in pregnant tissues from any stage (P7 through term), and therefore roughly paralleled those seen in response to 4-AP in nonpregnant and pregnant myometrium (see Figure 7). Phrixotoxin-2-elicited contractions in nonpregnant myometrium were on average significantly smaller than those seen in response to 4-AP; this finding leads us to suggest that phrixotoxin-2 may be blocking only a small amount of total outward (potassium) current, and that it is therefore capable of eliciting only a small depolarisation (and consequently contraction) in uterine smooth muscle cells.

### Kv4.3 expression, 4-AP-induced contraction time courses

By means of contraction experiments performed on tissues taken from animals at various stages of pregnancy (P7, P10, P15, and P20) we were able to establish time courses of the loss of both 4-AP responsiveness and Kv4.3 expression. At the earliest gestational point examined (P7) contractile responses to 4-AP were reduced compared to those seen in nonpregnant tissues (Figure 7.a), and at 300 μM 4-AP (the concentration at which contractile responses become maximal in nonpregnant myometrium), mean contractile responses to 4-AP in samples taken from mice from each stage of gestation (P7, P10, P15, and P20) were significantly reduced compared to those found in nonpregnant samples (Figure 7.a). Comparison by densitometry revealed a significant (*p = *0.01) reduction in Kv4.3 band densities in term-pregnant (P20) *vs*. either nonpregnant or P7 through P15 animals (Figure 7.b). The time course of changes in Kv4.3 expression was different from the time course of changes in 4-AP responses, as expression of Kv4.3 in mouse myometrium was significantly reduced only after the 15^th ^day of pregnancy (Figure 7.b). While there was variability in the level of expression of Kv4.3 at different stages of pregnancy, expression of Kv1.4 was found to be highly consistent throughout the stages of pregnancy and from experiment to experiment (Figure 7.b). Visceral smooth muscle (γ) actin expression was relatively constant until P20, at which point its expression was significantly increased, a finding which strongly supports those reported by Shynlova et al. [22].

## Discussion

In this study we demonstrate that the Kv channel blocker 4-AP potently contracts the mouse myometrium, and that it does so by a nerve- and endometrium-independent mechanism. Contractions elicited by 4-AP did not require functioning BK channels, and we found no evidence for a role for BK in the regulation of spontaneous contractility in either nonpregnant (Figure 2) or pregnant (data not shown) mouse myometrium. Remarkably, contractile responses to 4-AP disappear in pregnant mouse myometrium, to the point where 4-AP is unable to elicit any contraction at all in late pregnancy. A number of types of Kv channel α subunit proteins were found to be expressed in both pregnant and nonpregnant mouse myometrium, and one of these – Kv4.3 – was found to disappear from the mouse myometrium by the end of gestation, as was the contractile response to the Kv4.2/Kv4.3-specific blocker phrixotoxin-2. These data are consistent with the hypothesis that 4-AP contracts nonpregnant mouse myometrium by blocking Kv4.2 and/or Kv4.3, and that the disappearance of Kv4.3 in pregnancy accounts for the observed loss in 4-AP responses in this tissue.

Contractions in nonpregnant mouse myometrium occurred in response to surprisingly low concentrations of 4-AP (compared to those required to elicit contraction in other smooth muscles[17]), a finding perhaps best explained by the large number of different Kv channel subtypes we now report to be present in myometrium from both nonpregnant and term-pregnant mice (see Figure 5) including several types (e.g. Kv1.4, Kv4.2, and Kv4.3) known from the work of others to be sensitive to 4-AP [23]. Contractions in response to 4-AP have been shown to occur in a variety of non-uterine smooth muscles, including those found in the vasculature [24,25]. and gastrointestinal system [26-29]. In rat myometrium, 4-AP has recently been found to cause contractions in nonpregnant and, in contrast to our present findings in mouse, pregnant myometrium [21], although the concentrations of 4-AP employed in the rat experiments were significantly higher than those used in the present study. Mechanistically, 4-AP is thought to cause smooth muscle contraction by blocking one or more members of the Kv channel family, something 4-AP has long been known to be capable of doing in non-muscle cells [17]. 4-Aminopyridine has been shown to depolarise rat myometrium [30], and we assume that contractions elicited by 4-AP in myometrium are associated with membrane depolarisations as well.

In an effort to narrow our search for the molecular target(s) for 4-AP in myometrium we have been able to rule out several possibilities, including nerves, the endometrium, and BK channels. With respect to the latter, there appears to be a relatively high level of BK channel expression in myometrium in a number of species [11], and the expression of BK may be regulated by the hormonal environment and/or reproductive state of the animal [31]. A role for BK channels in the repolarisation of uterine myocyte membranes has been well-established [10,11,32]. It has also been suggested that BK channels may regulate the resting membrane potential of (and therefore the spontaneous contractile activity in) human and rat uterine myocytes [10]; as mentioned earlier, we have found no evidence of a role for BK channels in the regulation of spontaneous contractility in mouse myometrium, as we have found no effect of BK blockers such as TEA, verruculogen, or iberiotoxin on spontaneous contractility in myometrium from either pregnant or nonpregnant mice. In this our work supports that of Aaronson et al. [21], who argue that BK does not play a role in the regulation of contractility in the rat myometrium, and that one or more Kv channels are instead responsible for this regulation[21]. Since we now find that contractile responses to 4-AP in nonpregnant mouse myometrium (Figure 4) occur at concentrations significantly below those thought necessary to block BK channels in other tissues [17], and that pretreatment of nonpregnant mouse myometrium with concentrations of iberiotoxin or verruculogen which should be effective at blocking BK channels [33,34] had no effect upon subsequent contractile responses to 4-AP (Figure 2), we do not believe that 4-AP is contracting mouse myometrium by blocking BK channels. It therefore appears that the mechanism by which resting membrane potential in myometrial cells is set has yet to be determined.

The relatively high potency of 4-AP in nonpregnant mouse myometrium, coupled with our finding of the expression of a number of 4-AP-sensitive Kv channel α subunit proteins, suggests the blockade of Kv channels as a possible mechanism for 4-AP-elicited contractions in this tissue. While Kv channels are thought to be primarily responsible for limiting and reversing membrane depolarisation during the action potential [17,35], there is evidence that Kv channels can regulate resting membrane potential of smooth muscle cells in pulmonary artery [36-38], coronary artery [25], and airways [39]. There is, however, little evidence that Kv channels are active (and therefore available for blockade) at membrane potentials such as those found in resting, nonpregnant myometrium (-55 mV). However, a unique electrical property of myometrium may explain how Kv channels could regulate resting membrane potential in this tissue. Myometrium is apparently alone among smooth muscles in expressing a transient outward (A-type) current with a threshold of activation that is equal if not negative to the resting membrane potential [40,41]; this A-type current is known, in many cases, to be conducted by 4-AP-sensitive Kv channels, including Kv4.3. It is possible that the myometrium expresses a unique complement of Kv channels with distinctive biophysical properties that result in a 4-AP-sensitive current which is active at rest and therefore available for blockade by 4-AP. Interestingly, it is also known that a 4-AP-sensitive, A-type current present in nonpregnant rat myometrium disappears during pregnancy [19]; it may be the loss of this current which renders the term-pregnant mouse myometrium insensitive to 4-AP, as shown in the present work. It is also possible that the loss in A-type current expression accounts for the gradual movement of resting membrane potential to more positive values which is known to occur in the myometrium of women and rats during pregnancy [3].

We demonstrate the expression in mouse myometrium of a number of α subunit proteins belonging to the Kv family of potassium channels. While our preparations are highly enriched for uterine smooth muscle, we cannot rule out a minor contribution by nervous or vascular tissues to our findings of Kv channel α subunit proteins; histological examination of our preparations of uterine smooth muscle demonstrates, however, that the vast majority (> 95%) of the cells present in our preparations are uterine smooth muscle cells (data not shown), and thus the proteins present in our Western blot figures almost certainly reflect primarily the expression of proteins in these predominant (visceral smooth muscle) cell types. While there appear to be slight differences in the expression levels of some of the Kv channel α subunits shown in Figure 5 (e.g. there appears to be a little more Kv1.1 expression in term-pregnant myometrium than in nonpregnant tissues), we are unwilling at present to ascribe any significance to these differences other than the profound reduction in Kv4.3 expression which occurs near the end of gestation (Figures 5 and 7), a finding which is consistent with those of Song et al. [19] who found a reduction in Kv4.3 mRNA and protein expression in pregnant rat uterus which appears to be dependent upon estrogen.

We do not know with certainty that 4-AP causes contractions in nonpregnant mouse myometrium by blocking Kv channels, nor can we claim that the disappearance of Kv4.3 channels at the end of pregnancy is clearly responsible for the loss of 4-AP responsiveness seen in these tissues. However, our contraction studies with phrixotoxin-2 suggest that Kv4.2 and/or Kv4.3 could well be molecular targets for 4-AP in mouse myometrium, and since both phrixotoxin-2- and 4-AP-elicited contractions disappear in pregnant myometrium, we suggest that changes in Kv4.3 expression may result in functional changes in term-pregnant myometrium, with 4-AP responsiveness being at the very least an indicator of these changes. These observations are consistent with the hypothesis that the pregnant myometrium is by default a highly contractile organ, and that it is only the presence of a large amount of outward (polarising) potassium current that maintains the myometrium in a quiescent state until it is time for labour to begin; myometrium possesses, after all, a strong myogenic response to stretch, and certainly the uterus stretches to a remarkable degree in order to accommodate the developing foetus during pregnancy. Our functional and molecular results suggest that the disappearance of specific Kv channels (such as Kv4.3) may be responsible for a loss of outward (relaxing) potassium current and consequently the increase in contractility characteristic of labour.

Examination of the data contained within Figure 7 reveals a difference between the time courses of reductions in Kv4.3 expression and 4-AP responsiveness, with the loss of 4-AP responsiveness preceding the reduction in Kv4.3 expression in pregnant mouse myometrium by at least 2 weeks. Given that Kv channels are known to form heteromultimers of α and β subunits (as well as combine with various accessory proteins specific for Kv channels) which can result in a variety of different pharmacological and biophysical channel properties [42], it is possible that changes in β subunit and/or accessory protein expression could produce reductions in Kv4.3 channel function in pregnant myometrium without any changes in channel expression, and this could explain the difference in 4-AP and Kv4.3 time courses in pregnant myometrium. It is, however, also possible that fully-functional Kv4.3 channels are expressed in pregnant myometrial cells but are not present in sufficient amounts in the plasma membrane (e.g. they may be internalized to intracellular domains), rendering them insensitive to blockade by 4-AP and phrixotoxin-2.

## Conclusion

We have uncovered a significant difference in responsiveness to the Kv channel blockers 4-AP and phrixotoxin-2 between nonpregnant and pregnant mouse myometrium, and provide evidence for the expression of a number of Kv channel α subunit proteins in myometrium from both pregnant and nonpregnant mice; we also provide evidence of a profound reduction in Kv4.3 α subunit expression in term-pregnant mouse myometrium. Taken together these findings suggest a role for Kv channels in the regulation of uterine contractions, as well as a possible molecular basis for the functional changes which occur in the pregnant myometrium.

## Competing interests

The author(s) declare that they have no competing interests.

## Authors' contributions

R.B. performed almost all experiments in this study, and participated in a significant way in the design of experiments and analysis of the data; he was also heavily involved in the writing of this report. M.M. performed a number of the Western blot experiments, and M.S. performed a number of the contractile experiments. P.A. contributed to the design of the overall experimental approach. M.B. was responsible for the design and analysis of experiments, the overall questions being addressed, and the writing of this report, and also participated in a number of the actual experiments. All authors read and approved the final manuscript.
